# Potential Epigenetic Regulation in the Germinal Center Reaction of Lymphoid Tissues in HIV/SIV Infection

**DOI:** 10.3389/fimmu.2018.00159

**Published:** 2018-02-01

**Authors:** Xiaolei Wang, Huanbin Xu

**Affiliations:** ^1^Tulane National Primate Research Center, Tulane University School of Medicine, Covington, LA, United States

**Keywords:** Epigenectic regulation, germinal center reactions, follicular CD4 T helper cells, B cells, HIV

## Abstract

The production of high-affinity and broadly neutralizing antibodies plays a key role in the defense against pathogens. These antibody responses require effective germinal center (GC) reaction within anatomical niches of GCs, where follicular helper T (Tfh) cells provide cognate help to B cells for T cell-dependent antibody responses. Emerging evidences indicate that GC reaction in normal state and perhaps establishment of latent Tfh cell reservoir in HIV/SIV infection are tightly regulated by epigenetic histone modifications, which are responsible for activating or silencing chromatin. A better understanding of the mechanisms behind GC responses at cellular and molecular levels thus provides necessary knowledge for vaccination and immunotherapy. In this review, we discussed the epigenetic regulation of GC responses, especially for GC B and Tfh cell under normal state or HIV/SIV infection.

## Introduction

B-cell lineage commitment develops in primary lymphoid tissues such as fetal liver and bone marrow, and enters circulation (1). In secondary lymphoid tissues [such as lymph nodes, spleen, and gut-associated lymphoid tissues (GALT)], antigen-activated B cells experience clonal expansion, somatic hypermutation (SHM), and selection, and ultimately differentiate into antigen-specific memory subsets and plasma cells, which require T cell-dependent interactions for full responses (2, 3). Of these, germinal center (GC) reaction is the critical checkpoint in the development of T-dependent B-cell responses against foreign pathogens. Emerging studies have shown GC responses are strictly regulated by epigenetic modifications, which cooperate with timely expression of transcriptional factors for follicular B/T helper cell differentiation, thereby modulating antibody responses to foreign- and self-antigens (4). Therefore, understanding the intrinsic mechanisms involved in GC responses, and their dysregulation in HIV infection provides potential for the development of improved vaccines and immunotherapy.

## GC Formation and Reaction in Interaction Between GC B and Follicular Helper T (Tfh) Cells

Germinal centers are unique highly organized structures that formed within organized lymphoid tissues of both peripheral and mucosal (GALT) lymphoid tissues in response to T cell-dependent antigen. In GCs, Ag-activated B-cell clones proliferate and undergo SHM and selection, eventually produce antibodies with high-affinity and antigen specificity (5–7). For example, early GCs can first be histologically observed in mice at day 4 after immunization, in which B cells expand and differentiate into B cell blasts within the network of follicular dendritic cells (FDC) in the center of the follicle (5, 8). The dark zone (DZ) and light zone (LZ) in GCs could be microscopically distinguishable in lymphoid tissues. The DZ B cells (called centroblasts) highly proliferate, with opportunity to produce random immunoglobulin gene hypermutation and diversify Ig repertoire against foreign antigens. These DZ B cells leave DZ, and then migrate to the LZ, form LZ B cells (known as centrocytes), which are subject to clonal selection and terminal differentiation into memory B cells and plasma cells by signals from Tfh cells and FDCs. GCs are major sites for humoral immune responses, including B-cell development, differentiation and maturation, production of high-affinity antibodies that recognize and/or neutralize infectious pathogens.

The GC reaction is responsible for T-dependent humoral immune responses and is defined as the sequential process of B-cell differentiation, activation, maturation, resulting in antibody affinity maturation, and terminal differentiation, all that occurring within the GCs of lymphoid tissues. GC B cells undergo random SHM, Ig gene rearrangement, and clonal selection and eventually differentiate into long-lived memory B cells and high-affinity antibody-secreting plasma cells (8–12). By B cell receptor signaling *via* antigen binding, naïve B cells are initially activated and then migrate to the interfollicular (IF) region, where they interact with antigen-specific T cells and are thoroughly activated (13–15). These GC B founders express intermediate levels of BCL6 prior to follicular entry and GC seeding, and subsequent transit to the BCL6high state in B-cell commitment to the GC lineage, lagging behind Tfh migration into the follicle interior (16). The transcriptional repressor BCL6 is indispensable for GC B cell differentiation, repressing expression of the transcriptional factors IRF4/Blimp1 and formation of short-term antibody-secreting cell (ASC) (8, 17). However, only a proportion of these antigen-activated B cells are able to enter the GC zones and participate in the GC reaction (8). A subset of activated B cells in the IF zones at the peripheral follicles could differentiate into ASCs, which produce low-affinity antibodies to pathogens, albeit with a rapid antibody responses (18). Another pool of antigen-specific GC B cells with the highest relative affinity gains access to the lymphoid follicles, aggregated to form GCs (19–22). Within anatomical niches of mature GCs, GC B cells in the DZ (densely packed blasts, centroblasts) rapidly proliferate, undergo random SHM catalyzed by activation-induced cytidine deaminase (AID), and rearrange and diversify their IgV genes, resulting in mutant GC B cell clones with a broader repertoire of antibody specificity (23–25). Upon transition into the LZ (sparsely populated B cells, centrocytes), GC B cells with the highest affinity B cell receptors are positively selected by GC Tfh cells. Signaling from GC Tfh cells, such as CD40, IL-4, IL-9, IL-21, and ICOS, plays a pivotal role in the GC reaction during intermittent cognate engagement between GC B and Tfh cells (26–28). Rapid interactions between GC B and Tfh cells in DZ/LZ occur, as indicated by fluctuating CXCR4 and/or CXCR5 expression, which facilitate several reiterative rounds of B cell mutation and selection, resulting in terminal differentiation into highly specific memory B cells and plasma cells (5, 7, 11, 29). In the GC reaction, increasing evidence indicates that Ig SHM and selection of antigen-experienced B cells are needed for development of broadly neutralizing antibodies at checkpoints during B cell activation (30).

## Epigenetic Histone Regulation and Its Potential in B-Cell Differentiation and Antibody Responses

Epigenetic alteration at posttranslational modification (PTM) is able to regulate gene expression or repression, and control cellular function without genomic sequences changes (4). Epigenetic histone modification, either by adding or removing histone methylation, acetylation, phosphorylation, or ubiquitination at histone posttranslational levels, alters chromatin structure and represses (such as chromosomal condensation) or promotes target gene transcriptional pathways affecting cell development, differentiation, and cell fates, and thereby modulates cell functions in both programmed development, or in response to disease states (31). Under the control of epigenetic regulation, cell commitment to a specific differentiated lineage involves the activation of specific genes while maintaining the other gene silence at the genomic loci (32). Among various chromatin-modifying epigenetic factors, polycomb G (PcG) proteins act in multimeric complexes known as polycomb repressive complexes (PRCs, including PRC1, PRC2, and PhoRC), which are specifically involved in histone PTMs. PRC2, composed of three subunits [enhancer of zeste homolog 2 (EZH2)/EZH1, SUZ12, and EED], binds to specific targets of chromatin, and then the enzymatic subunit EZH2 catalyzes the di- and tri-methylation of Lys 27 residues on histone H3 to generate H3K27me2/3 (33), which mediates changes in chromatin structure, transcriptional repression, somatic processes during embryonic development, lineage commitment, and even tumorigenesis (34–41). H3K27me3 could recruit PRC1 (BMI1 subunit) (42, 43), and thus stabilize polycomb G-mediated repression (39, 44, 45). EZH2 is a central core component of the PcG family, as it serves as histone-lysine *N*-methyltransferase to catalyze H3-K27 methylation (13). Conversely, aberrant EZH2 overexpression and subsequent SHMs are associated with cancer occurrence (13, 46, 47). Although EZH2 is directly responsible for the trimethylation of H3-K27, EZH2 overexpression does not directly increase H3K27me3, but instead results in PRC4-mediated H1K26 trimethylation, upregulation of demethylase (JMJD3/UTX), and phosphorylation of Ezh2 (P-Ezh2-Ser21) (48–51). Loss of H3K27me3 despite high EZH2 and demethylase levels is thus believed to be due to transcriptional suppression of H3K27me3-target genes by increased demethylase or other unknown mechanisms. The degree of lysine methylation within histones (mono-, di-, and tri-) is one modification with distinctive nuclear features and transcriptional states of target genes, and a major determinant for genome organization. Both lysine methyltransferases (KMTs) and lysine demethylases (KDMs) have specificity for specific lysine residues and degrees of methylation within the histone tails. Lysine (K) motifs within the histone tails are primary sites to recruit chromatin-modifying enzymes such as methyltransferase, leading to specific gene repression or activation (52). For examples, H4K20 and H3K27 monomethyation (H4K20/27me1) is associated with active promoters, while H4K20 and H3K27 trimethylation (H4K20m/27me3) is affiliated with gene repression and compacted genomic regions. However, H3K4me3 is generally responsible for active chromatin (53, 54). H3K27me2 shows a similar distribution and role to H3K27me3 (55, 56). In addition, histone demethylation/acetylation, respectively, catalyzed by demethylase UTX/JMJD3 (H3K27me2/3 substrate), LSD1 (H3K4me2 substrate), JMJD2 (H3K9me3 substrate), JARID (H3K4me3 substrate), or acetyltransferase, is also associated with active transcription, antagonizing the repression of gene expression induced by H3K27me2/3 (57, 58).

**Figure 1:**
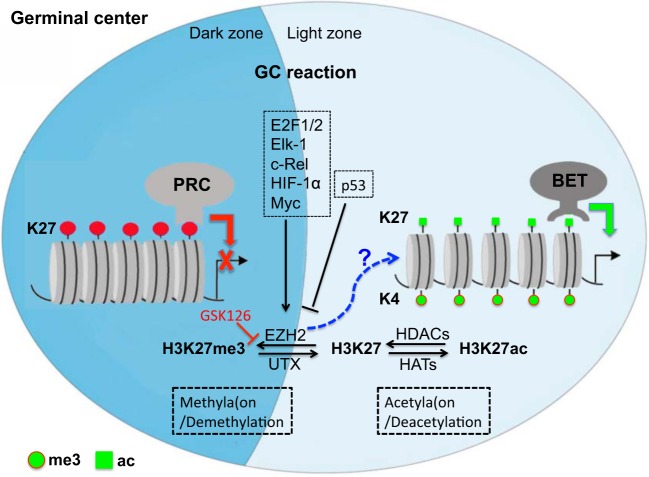
Repressive and active gene regulation in germinal center (GC) reaction by epigenetic histone modification at posttranslation level in GCs. Epigenetic regulation, histone methylation/demethylation, or/and acetylation/deacetylation, is involved in B cell development, GC formation, somatic hypermutation, and Ab affinity maturation. Note that methyltransferase EZH2 is highly expressed in GC B cells (see our preliminary study), but its unique target mark-H3K27me3 is undetectable in developing neonates, suggesting that EZH2 may regulate the GC reaction *via* alternative mechanisms or balanced by histone demethylation/acetylation. PRC, polycomb repressive complex; EZH2, enhancer of zeste homolog 2; UTX, X chromosome-encoded histone demethylase; HATs, histone acetyltransferases; HDACs, histone deacetylases; BET, bromodomain and extra-terminal motif protein.

In the context of antibody responses, B-cell development and the GC reaction is precisely fine-tuned by histone modifiers (59). Specifically, epigenetic modification controls B-cell differentiation and maturation, thereby regulating Ab responses (4, 13, 60–64). Upon activation by antigens, GC B cells upregulate and highly express EZH2, which segregates primarily in either the LZ or/and DZ (60, 65), and plays a pivotal role in B cell differentiation, GC formation, normal immunoglobulin VDJ recombination, inhibition of terminal B-cell differentiation, and lymphomagenesis *via* histone trimethylation (H3K27me3) (13, 61, 63, 66). High expression of EZH2, cooperating with Bcl6, is required to maintain the GC B cell phenotype but its relevance diminishes concomitant with GC B cells exiting GCs and terminal differentiation (upregulated IRF4 and BLIMP1), suggesting an important role for this protein in maintaining B cell division (8, 61, 67). EZH2 depletion or mutation perturbs B-cell differentiation and GC reaction with reduction in high-affinity antibodies, while overexpression of EZH2 promotes lymphomagenesis (63, 66). These findings suggest that EZH2 is essential for normal B-cell differentiation, activation, as well as maturation. Additionally, expression of EZH2 is also precisely regulated in various physiological and pathogenic processes (13, 68). Factors, including c-Rel, E2F1/2, Elk-1, and HIF-1α directly bind to the EZH2 promoter, leading to EZH2 expression (69–72). For example, c-Rel supports GC B cell proliferation and maintains the GC through upregulation of EZH2. Another factor, Myc could also indirectly induce EZH2 expression through miRNA or retinoblastoma protein-E2F (pRB-E2F) (73). Myc also enables GC B cell division and transformation, as Myc+ GC B cells are highly proliferative cell subsets (12, 74, 75), compared with p53-mediated suppression of EZH2 expression (76). Combined, multiple *B cell-intrinsic epigenetic alterations* may be involved in instructing B cells to undergo B cell development, GC formation, SHM, and Ab affinity maturation in the GC reaction, including differentiation to memory B cells or long-lived plasma cells (8, 12, 63, 66) (Figure 1).

## Epigenetic Regulation in Tfh Cell Reservoirs in HIV/SIV Infection

**Figure 2:**
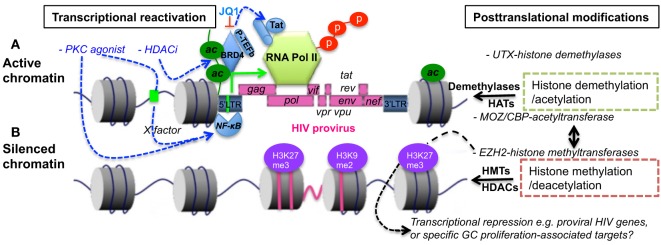
Epigenetic regulation in transcriptional reactivation **(A)** or repression **(B)** of proviral transcription. Histone acetylation or/and specific demethylation leads to a less compact chromatin and active transcription. In comparison, histone deacetylation or/and specific methylation causes condensed and transcriptionally silenced chromatin. BRD4 binds to chromatin *via* acetylated histone, blocking histone deacetylation and recruiting P-TEFb that initiates phosphorylation of polymerase II. BET bromodomain inhibitor JQ1 disassociates BRD4/P-TEFb, resulting in recruitment of P-TEFb/Tat complexes to LTR promoter, and specific reactivation of HIV latency. PKC agonists or HDACi, which are regulators of gene expression that enzymatically remove the acetyl group from histone, facilitate transcriptional reactivation of provirus. *BET*, bromodomain and extra-terminal motif; HATs, histone acetyltransferases, e.g., MOZ or CBP; HDACs, histone deacetylases; HMTs, histone methyltransferases; *BRD4*, BET protein 4; P-TEFb, positive transcription elongation factor b; EZH2, enhancer of zeste homolog 2; UTX, X chromosome-encoded histone demethylase; CBP, CREB-binding protein; MOZ, monocytic leukemia zinc finger protein; PKC, proteinase kinase C; HDACi, histone deacetylase inhibitor.

CD4 T cells preferentially develop into Tfh cells following repetitive T cell receptor interactions and activation, and the proinflammatory cytokines produced during persistent viral infections (77–79). Notably, epigenetic regulation is also involved in T cell differentiation and memory formation (80–82). These epigenetic alterations include PTMs. For example, EZH2 restricts the differentiation of Th1 and Th2 cells *via* H3K27me3-mediated gene repression (83). Conversely, upregulation of UTX, an H3K27 demethylase supports Tfh cell differentiation and eliminates persistent viral infections (84). As indicated in Figure 2, epigenetic histone modification in virus-infected cells is implicated in the immune evasion and latency in HIV infection and AIDS (85–90). The reactivation of HIV latency could be regulated by epigenetic modification through effects on the chromatin state of the viral promoter in the LTR sequence (90–93). The BET (bromodomain and extraterminal domain) family, including BRD2, BRD3, BRD4, and BRDT, are important epigenetic regulators facilitating the gene transcription in chromatin (94). BRD4, a chromatin adaptor protein, forms a tight complex with chromatin through two tandem bromodomains (BD1 and BD2), acetylate lysine residues in histone 3 and 4 at both enhancer and general promoter regions of chromatin, recruiting positive transcription elongation factor-b (P-TEFb) (95). The latter facilitates cellular transcription by phosphorylating RNA polymerase II at the serine residue in the C-terminal domain (96). However, recent studies indicate that BET bromodomain inhibitor (e.g., JQ1) dissociates BRD4 from BRD4/P-TEFb complex, resulting in P-TEFb/HIV Tat recruitment to the LTR promoter and reactivation of HIV-infected cells (97, 98). Antiretroviral drugs, in combination with epigenetic regulatory agents, are promising to effectively reactivate HIV latency *via* histone deacetylase inhibitors (HDACi), histone methyltransferase inhibitors, or DNA methyltransferase inhibitors.

As described above, GC Tfh cells provide help for optimal B-cell differentiation, and antibody affinity maturation (10, 99). Interactions of GC B cells with GC Tfh cells are critical for antibody production. However, persistent SIV infection leads to aberrant GC Tfh cell expansion, ultimate depletion, abnormal B-cell responses, and viral reservoir establishment as a major source of the HIV reservoir within sanctuary sites in lymphoid tissues (79, 100, 101), consistent with the facts that organized lymphoid tissues represent the major tissue reservoir for HIV replication and latency (102–104), even during prolonged ART (78, 105–109). Although studies in adults indicate that HIV infection leads to abnormal B-cell and Tfh cell responses (110–115), yet, studies on the regulation of B-cell responses, especially at the cellular and molecular levels within GCs, needed to be further investigated in HIV/SIV infection.

## Author Contributions

XW wrote manuscript and HX revised the manuscript.

## Conflict of Interest Statement

The authors declare that the research was conducted in the absence of any commercial or financial relationships that could be construed as a potential conflict of interest. The reviewer KT and handling editor declared their shared affiliation.
